# Supplementary material for: Comparison of the Micronaut-AM System and the EUCAST Broth Microdilution Reference Method for MIC Determination of Four Antifungals against Aspergillus fumigatus
Source: J Fungi (Basel). 2023 Jun 30;9(7):721. doi: 10.3390/jof9070721 (PMC10381152; doi:10.3390/jof9070721)
Supplement: Supplementary file 1 [file jof-09-00721-s001.zip › jof-2392758-supplementary.pdf]

Table S1. Comparison of MICs (mg/L) and categories for 61 azole-susceptible isolates.

N° isolate	Voriconazole			Itraconazole			Posaconazole		
	MIC (mg/L)		C	MIC (mg/L)		C	MIC (mg/L)		C
	E	M	E/M	E	M	E/M	E	M	E/M
**1**	0.25	0.0625	S/S	0.25	0.03125	S/S	0.125	0.015625	S/S
**2**	0.25	0.0625	S/S	0.25	0.0625	S/S	0.25	0.015625	S/S
**3**	0.25	0.0625	S/S	0.25	0.0625	S/S	0.125	0.015625	S/S
**4**	0.5	0.125	S/S	0.125	0.0625	S/S	0.0625	0.03125	S/S
**5**	0.25	0.0625	S/S	0.125	0.0625	S/S	0.125	0.015625	S/S
**6**	0.5	0.03125	S/S	0.5	0.03125	S/S	0.125	0.015625	S/S
**7**	0.25	0.0625	S/S	0.25	0.25	S/S	0.125	0.015625	S/S
**8**	0.5	0.03125	S/S	0.25	0.03125	S/S	0.125	0.015625	S/S
**9**	0.25	0.0625	S/S	0.25	0.03125	S/S	0.125	0.015625	S/S
**10**	0.25	0.03125	S/S	0.25	0.03125	S/S	0.125	0.015625	S/S
**11**	0.25	0.0625	S/S	0.25	0.03125	S/S	0.0625	0.015625	S/S
**12**	0.125	0.0625	S/S	0.0625	0.0625	S/S	0.0625	0.015625	S/S
**13**	0.5	0.0625	S/S	0.25	0.0625	S/S	0.25	0.03125	S/S
**14**	0.25	0.0625	S/S	0.25	0.03125	S/S	0.125	0.03125	S/S
**15**	0.125	0.03125	S/S	0.25	0.03125	S/S	0.125	0.015625	S/S
**16**	0.25	0.0625	S/S	0.25	0.03125	S/S	0.125	0.015625	S/S
**17**	0.5	0.03125	S/S	0.25	0.03125	S/S	0.125	0.015625	S/S
**18**	0.25	0.0625	S/S	0.25	0.0625	S/S	0.125	0.015625	S/S
**19**	0.5	0.125	S/S	0.25	0.0625	S/S	0.25	0.03125	S/S
**20**	0.125	0.03125	S/S	0.5	0.03125	S/S	0.125	0.0078125	S/S
**21**	0.125	0.03125	S/S	0.015625	0.03125	S/S	0.0625	0.015625	S/S
**22**	0.5	0.0625	S/S	0.25	0.0625	S/S	0.125	0.015625	S/S
**23**	0.25	0.0625	S/S	0.5	0.0625	S/S	0.125	0.03125	S/S
**24**	0.5	0.03125	S/S	0.25	0.03125	S/S	0.25	0.015625	S/S
**25**	0.25	0.03125	S/S	0.25	0.03125	S/S	0.125	0.015625	S/S
**26**	0.25	0.03125	S/S	0.25	0.03125	S/S	0.125	0.015625	S/S
**27**	0.25	0.03125	S/S	0.25	0.0625	S/S	0.25	0.0078125	S/S
**28**	0.25	0.0625	S/S	0.125	0.03125	S/S	0.125	0.015625	S/S
**29**	0.25	0.015625	S/S	0.25	0.03125	S/S	0.125	0.015625	S/S

**30**	0.125	0.0625	S/S	0.5	0.0625	S/S	0.125	0.03125	S/S
**31**	0.125	0.015625	S/S	0.25	0.03125	S/S	0.125	0.015625	S/S
**32**	0.25	0.03125	S/S	0.25	0.0625	S/S	0.125	0.015625	S/S
**33**	0.25	0.0625	S/S	0.5	0.0625	S/S	0.125	0.015625	S/S
**34**	0.25	0.0625	S/S	0.25	0.125	S/S	0.25	0.015625	S/S
**35**	0.25	0.0625	S/S	0.25	0.0625	S/S	0.125	0.015625	S/S
**36**	0.25	0.0625	S/S	0.25	0.0625	S/S	0.125	0.03125	S/S
**37**	0.25	0.0625	S/S	0.25	0.0625	S/S	0.125	0.015625	S/S
**38**	0.0625	0.015625	S/S	0.125	0.03125	S/S	0.125	0.0078125	S/S
**39**	0.5	0.03125	S/S	0.25	0.03125	S/S	0.125	0.015625	S/S
**40**	0.25	0.03125	S/S	0.125	0.0625	S/S	0.125	0.015625	S/S
**41**	0.25	0.03125	S/S	0.25	0.0625	S/S	0.125	0.015625	S/S
**42**	0.5	0.125	S/S	0.25	0.0625	S/S	0.25	0.015625	S/S
**43**	0.125	0.03125	S/S	0?5	0.125	S/S	0.125	0.015625	S/S
**44**	0.25	0.0625	S/S	0.25	0.03125	S/S	0.125	0.015625	S/S
**45**	0.25	0.0625	S/S	0.25	0.125	S/S	0.25	0.015625	S/S
**46**	0.25	0.03125	S/S	0.25	0.0625	S/S	0.25	0.015625	S/S
**47**	0.25	0.0625	S/S	0.5	0.0625	S/S	0.125	0.015625	S/S
**48**	0.25	0.0625	S/S	0.25	0.125	S/S	0.125	0.015625	S/S
**49**	0.25	0.03125	S/S	0.5	0.03125	S/S	0.125	0.0078125	S/S
**50**	0.25	0.0625	S/S	0.5	0.03125	S/S	0.25	0.015625	S/S
**51**	0.25	0.0625	S/S	0.25	0.0625	S/S	0.125	0.03125	S/S
**52**	0.25	0.03125	S/S	0.25	0.03125	S/S	0.125	0.015625	S/S
**53**	0.5	0.0625	S/S	0.25	0.0625	S/S	0.25	0.015625	S/S
**54**	0.5	0.03125	S/S	0.25	0.03125	S/S	0.25	0.0078125	S/S
**55**	1	0.25	S/S	0.25	0.5	S/S	0.25	0.0625	S/S
**56**	0.25	0.0625	S/S	0.25	0.125	S/S	0.125	0.015625	S/S
**57**	0.25	0.125	S/S	0.5	1	S/S	0.125	0.0625	S/S
**58**	0.5	0.0625	S/S	0.25	0.25	S/S	0.25	0.03125	S/S
**59**	0.25	0.5	S/S	0.125	0.5	S/S	0.125	0.1125	S/S
**60**	0.5	0.25	S/S	0.5	0.5	S/S	0.25	0.125	S/S
**61**	0.25	0.03125	S/S	0.25	0.03125	S/S	0.125	0.015625	S/S

MIC: Minimum Inhibitory Concentration, C: Category, E: EUCAST, M: Micronaut-AM, S: susceptible.
